# Supplementary material for: Structural basis of BAK activation in mitochondrial apoptosis initiation
Source: Nat Commun. 2022 Jan 11;13:250. doi: 10.1038/s41467-021-27851-y (PMC8752837; doi:10.1038/s41467-021-27851-y)
Supplement: Supplementary file 1 — Supplementary information [file 41467_2021_27851_MOESM1_ESM.pdf]

Supplementary information

Structural Basis of BAK Activation in Mitochondrial Apoptosis Initiation

Geetika Singh[1,2], Cristina D. Guibao[1,2], Jayaraman Seetharaman[1], Anup Aggarwal[1,2], Christy R. Grace[1], Dan E. McNamara[1,2], Sivaraja Vaithiyalingam[1], M. Brett Waddell[1], Tudor Moldoveanu[1,2,*]

[1]Department of Structural Biology
[2]Department of Chemical Biology and Therapeutics
St. Jude Children's Research Hospital, Memphis, TN, USA

*To whom correspondence should be addressed: tudor.moldoveanu@stjude.org

Supplementary Tables 1-4
Supplementary Figures 1-9
Uncropped gels and blots from Supplementary Figures

Supplementary Table 1. Kinetic, thermodynamic, and functional parameters for BH3 peptide binding and activation of BAK. Related to Figure 5 and Supplementary Figures 1, 4, 7

	Isothermal titration calorimetry (ITC)				Surface plasmon resonace (SPR)				Liposome permeabilization
**BH3:BAK complexes**	**ΔG (kcal/mol)**	**K_D (μM)**	**ΔH (kcal/mol)**	**-TΔS (kcal/mol)**	**k_a (M^{-1} s^{-1})**	**k_d (s-1)**	**K_D (μM)**	**Rmax (RU)**	**EC50 (nM)**
WT BAK BH3: WT BAK	X	X	X	X	X	X	67±1	26.7±0.2	35.8±30.4[a]
WT BID BH3: WT BAK	-6.34±0.04	22.70±1.56	-6.75±0.07	0.41±0.11	4.19±0.02 ×10^3	2.61±0.01 ×10^{-1}	62.3±0.4	26.2±0.1	59±12[b]
M(3)W(5) BID: WT BAK	-8.41±0.07	0.69±0.09	-9.9±1.1	1.49±1.18	6.21±0.01 ×10^4	3.14±0.01 ×10^{-2}	0.506±0.001	77.6±0.1	79±49[b]
W(3)W(5) BID: WT BAK	-9.02±0.10	0.25±0.04	-14.55±1.76	5.53±1.6	7.19±0.01 ×10^4	2.41±0.01 ×10^{-2}	0.336±0.001	79.2±0.1	X

X, not determined

[a] EC50 for data in Supplementary Figure 4b

[b] EC50 for data in Supplementary Figure 7c

Supplementary Table 2. X-ray data collection and refinement statistics for BAK complexes.

PDB ID	W(3)W(5) BID BH3: BAK complex 7m5a	M(3)W(5) BID BH3-BAK complex 7m5b	BAK BH3-BAK complex 7m5c
Data collection			
Space group	I222	$P2_12_12_1$	$P2_1$
Cell dimensions			
a, b, c (Å)	65.86, 70.85, 71.39	50.19, 83.18, 110.03	89.39, 132.22, 89.38
α, β, γ (°)	90, 90, 90	90, 90, 90	90, 119.20, 90
Resolution (Å)	35.70 - 1.50 (1.56 - 1.50)[a]	37.08 - 1.85 (1.90 - 1.85)	29.34 – 3.06 (3.14-3.06)
Total reflections	185563	222410	86262
Unique reflections	26921 (2574)	39200 (1698)	30995 (1458)
R_{merge} (%)	4.8 (45.3)	3.7 (86.3)	10.2 (54.0)
I / σI	41.8 (2.4)	39.4 (1.1)	7.0 (1.3)
Completeness (%)	99.3 (97.8)	96.5 (85.2)	91.1 (58.7)
Redundancy	6.9 (5.2)	5.7 (4.2)	2.8 (2.1)
$CC_{1/2}$ (%)	99.5 (88.8)	100 (67.5)	99.0 (60.4)
Refinement			
Reflections used in refinement	26917 (2727)	35670 (2082)	30951 (1358)
R_{work} (%)	18.5 (25.5)	17.2 (30.7)	21.9 (31.8)
R_{free} (%)	21.9 (25.4)	20.3 (33.6)	25.3 (34.5)
Number of atoms			
Protein	1445	2900	13509
Ligand/ion		1	49
Water	141	304	
Average B-factor (Å^2)	22.12	24.52	62.96
Protein	21.05	23.51	62.93
Ligand/ion		17.35	71.73
Water	33.02	34.15	
R.m.s deviations			
Bond lengths (Å)	0.009	0.019	0.015
Bond angles (°)	0.98	1.33	1.82
Ramachandran Plot			
Favored (%)	100	100	99
Outliers (%)	0	0	1

[a]Statistics for the highest-resolution shell are shown in parentheses. Data for each structure was collected from a single crystal. See also Figures 1, 6, Supplementary Figures 2, 8, 10.

Supplementary Table 3. Electrostatic network stabilizing helix in crystal structures of BH3-bound BAK complexes. Related to Figures 1, 6, 7, Supplementary Figures 2, 8, 9.

Donor/Acceptor residue/helix/group	R42/ α1/ NH1	R42/ α1/ NH2	E46/ α1/ OE2	N86/ α2/ OD1	N86/ α2/ ND2	D90/ α2-α3/ OD1	D90/ α2-α3/ OD2	R137/ α5/ NE	R137/ α5/ NH1	R137/ α5/ NH2	R87/ α2/ NH1	R87/ α2/ NH2
Y38/α1/OH	3.0<br>A:2.6<br>C:2.6<br>E:2.6<br>G:2.6<br>I:2.7<br>K:2.6<br>M:2.6<br>O:2.6<br>Q:2.6<br>S:2.6<br>A:2.9<br>C:2.8<br>2.8	2.8<br>2.8<br>A:2.8<br>C:2.9<br>2.9			C:3.6							
R42/α1/NE			A:3.6<br>C:3.7<br>E:3.6<br>G:3.7<br>I:3.7<br>K:3.7<br>M:3.7<br>O:3.7<br>Q:3.7<br>S:3.7<br>A:2.8<br>C:2.9<br>3.0<br>A:2.8<br>C:2.8<br>2.8									
R42/α1/NH1			3.6<br>3.3	A:3.3<br>C:3.5								
R42/α1/NH2			3.3	A:3.2<br>C:3.2<br>3.2<br>A:3.2	A:2.8<br>C:2.5<br>E:2.8<br>G:2.8<br>I:nd<br>K:2.8<br>M:2.9<br>O:2.8<br>Q:2.8<br>S:nd<br>3.3<br>C:3.5<br>3.6	3.2	2.7					
E46/α1/OE1									3.5<br>T:3.7	3.5		
E46/α1/OE2					A:3.3<br>E:3.5<br>G:3.3<br>I:nd<br>K:3.5<br>M:3.4<br>O:3.3<br>Q:3.6<br>S:nd				2.8<br>A:2.8	3.4	A:2.9	A:3.6
N86/α2/OD1								A:3.4<br>E:3.5<br>G:3.5<br>I:nd<br>K:3.8<br>M:3.5<br>O:3.4<br>Q:3.5<br>S:nd	A:3.6	A:3.1<br>C:3.0	A:2.8	A:2.8
N86/α2/ND2										C:3.3		
D90/α2-α3/OD1								3.3	K:3.4<br>A:2.8<br>C:2.8<br>2.9<br>2.9<br>A:3.0<br>C:3.0<br>3.0	2.9<br>A:3.1<br>C:3.0<br>3.1<br>A:2.6<br>C:2.8<br>2.8		
D90/α2-α3/OD2								2.8				

Distances are color coded by crystal structure. Distances connecting helix α1 to the rest of the domain are bold and are summarized for each complex below. These numbers are also shown above each complex in Figures 1f, 5e, 5g, Supplementary Figures 2c, 2d, 7g, 7i, 9.

- Apo BAK (PDB ID: 2imt exhibits 1 distance ≤ 3.2 Å and 3 distances ≤ 3.6 Å)
- BAK-BAK BH3 autoactivated covalent complex (PDB ID: 7m5c; distances within the individual chains of the 10 complexes in the asymmetric units are summarized; with exception of complexes IJ and ST, which have poor electron density in this region, and CD with only 1 distance ≤ 3.2 Å, all complexes exhibit 1 distance ≤ 3.2 Å and 1 distance ≤ 3.6 Å; nd, electron density not defined)
- BAK-M(3)W(5) BID BH3 directly activated covalent complexes (PDB ID: 7m5b; both complexes in the asymmetric unit exhibit 1 distance ≤ 3.2 Å and 1 distance ≤ 3.6 Å shown according to chain)
- BAK:W(3)W(5) BID BH3 inactivated complex (PDB ID: 7m5a; 3 distances ≤ 3.2 Å; 1 distance ≤ 3.6 Å)
- BAK:BIM-RT directly activated complex (PDB ID: 5vwv; 1 distance ≤ 3.6 Å within the domain) and 2 distances ≤ 3.2 Å to TFA unnatural ligand modeled at the interface)
- BAK:BIM-RT directly activated complex (PDB ID: 5vww; chain A, 2 distances ≤ 3.2 Å; chain B, no electron density visible for side chains modeled at the interface)
- BAK:BIM-h3Pc inactivated complex (PDB ID: 5vwz; chain A, 1 distance ≤ 3.2 Å and 2 distances ≤ 3.6 Å within the domain, and 2 distances ≤ 3.2 Å to BIM P_c not shown in the table; chain C, 3 distances ≤ 3.6 Å within the domain, and 2 distances ≤ 3.2 Å to BIM P_c not shown in the table)
- BAK:BIM-h3Pc-RT inactivated complex (PDB ID: 5vwy; 1 distances ≤ 3.6 Å within the domain and 2 distances ≤ 3.2 Å to BIM P_c not shown in the table)

Supplementary Table 4. Oligonucleotide sequences

Construct		
pNIC28-Bsa4-MEAS-BAK	BAK_LIC_F<br>5'-tacttccaatccatggaagcttcagcttc-3'	BAK_LIC_R<br>5'-gtcatttccacctataataccattgcccaagttc-3'
pRETROX-mCherry-BAK	BAK_NcoI_F<br>5'-tatatataccatggcttcggggcaaggc-3'	BAK XhoI_R<br>5'-atatatatctcgagtcatgatttgaagaatcttcg-3'
pMX-BID-IRES-GFP	BID_BamHI_F<br>5'-gttaattaaggatccatgtgcagcggtgctgg-3'	BID_EcoRI_R<br>5'-gccagaaatgggatggactaacgggaattcctgcag-3'
Mutant Name	Forward Primer	Reverse Primer
BAK C166S	tcatgctgcatcacagcattgcccggtgg	agtacgacgtagtgtcgtaacgggccacc
BAK G184C	cagccctgaacttgtgcaatggtctcgagc	cgtcgggacttgaacacgttaccagagctcg
TEV site primer 1	ggagagcctgccctgcccgaaaacctgtctgcttctgaggagcagg	cctgctcctcagaagcagacaggttttcgggcagggcaggctctcc
TEV site primer 2	cctgcccctgcccgaaaacctgtacttccagtctgcttctgaggagcag	ctgctcctcagaagcagactggaagtacaggttttcgggcagggcagg
TEV site primer 3	cccgaaaacctgtacttccagagcggtacgtctgcttctgagg	cctcagaagcagacgtaccgctctggaagtacaggttttcggg
BAK M71A	aacctagcagcaccgcggggcaggtgggac	ttggatcgtcgtggcgccccgtccaccctg
BAK V74A	catggggcaggcgggacggcagc	gtaccccgtccgccctgccgtcg
BAK L78A	ggtgggacggcaggccgccatcatcggg	ccaccctgccgtccggcggtagtagccc
BAK I81A	ggcagctcgccatcgccggggacgacatca	ccgtcgagcggtagcggcccctgctgtagt
BAK I81R	acggcagctcgccatcagggggggacgac	tgccgtcgagcggtagtccccccctgctg
BAK D83A	cgccatcatcggggccgacatcaaccgac	gcggtagtagccccggctgtagttggctg
BAK I85A	catcatcggggacgacgccaaccgacgctatgac	gtagtagcccctgctgcggttggctgcgatactg
BAK I85R	catcatcggggacgacaggaaccgacgctatgactc	gtagtagcccctgctgtccttggctgcgatactgag
BAK Y89A	cgacatcaaccgacgcgctgactcagagttccag	gctgtagttggctgcgcgactgagtctcaaggtc
BAK F93A	gacgctatgactcagaggcccagaccatgttgcagc	ctgcgatactgagtctccgggtctggtacaacgtcg
BAK R137A	gggcttcggctacgctctggccctacac	cccgaagccgatgcgagaccgggatgtg
BAK R42A	ccgcagctacgtttttacgcccatcagcaggaac	ggcgtcgatgcaaaaaatgcgggtagtcgtccttg
BAK E46A	ccgccatcagcaggcacaggaggctgaag	ggcggtagtcgtccgtgtcctccgacttc

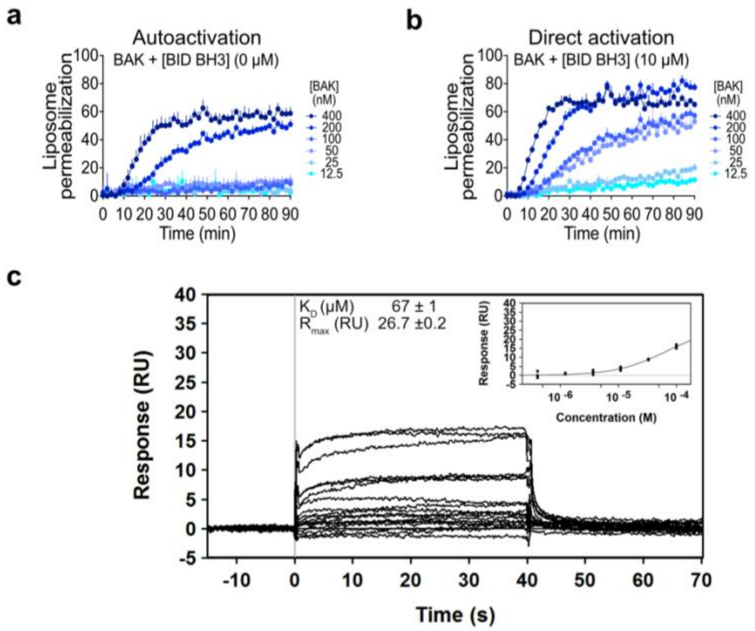

Supplementary Figure 1. Biochemical details of BAK autoactivation. Related to Figure 1, Supplementary Table 2. a and b, Autoactivation and direct activation of BAK in liposome permeabilization assays measuring kinetics of quenched fluorescent dye release. Liposome permeabilization is quantified as the percentage of fluorescent dye release normalized relative to permeabilization by 3% CHAPS detergent. Data are presented as mean + SD for one representative of n=4 experiments each of n=3 technical replicates. **c,** Surface plasmon resonance (SPR) sensorgrams of BAK titration with BAK BH3 peptide.

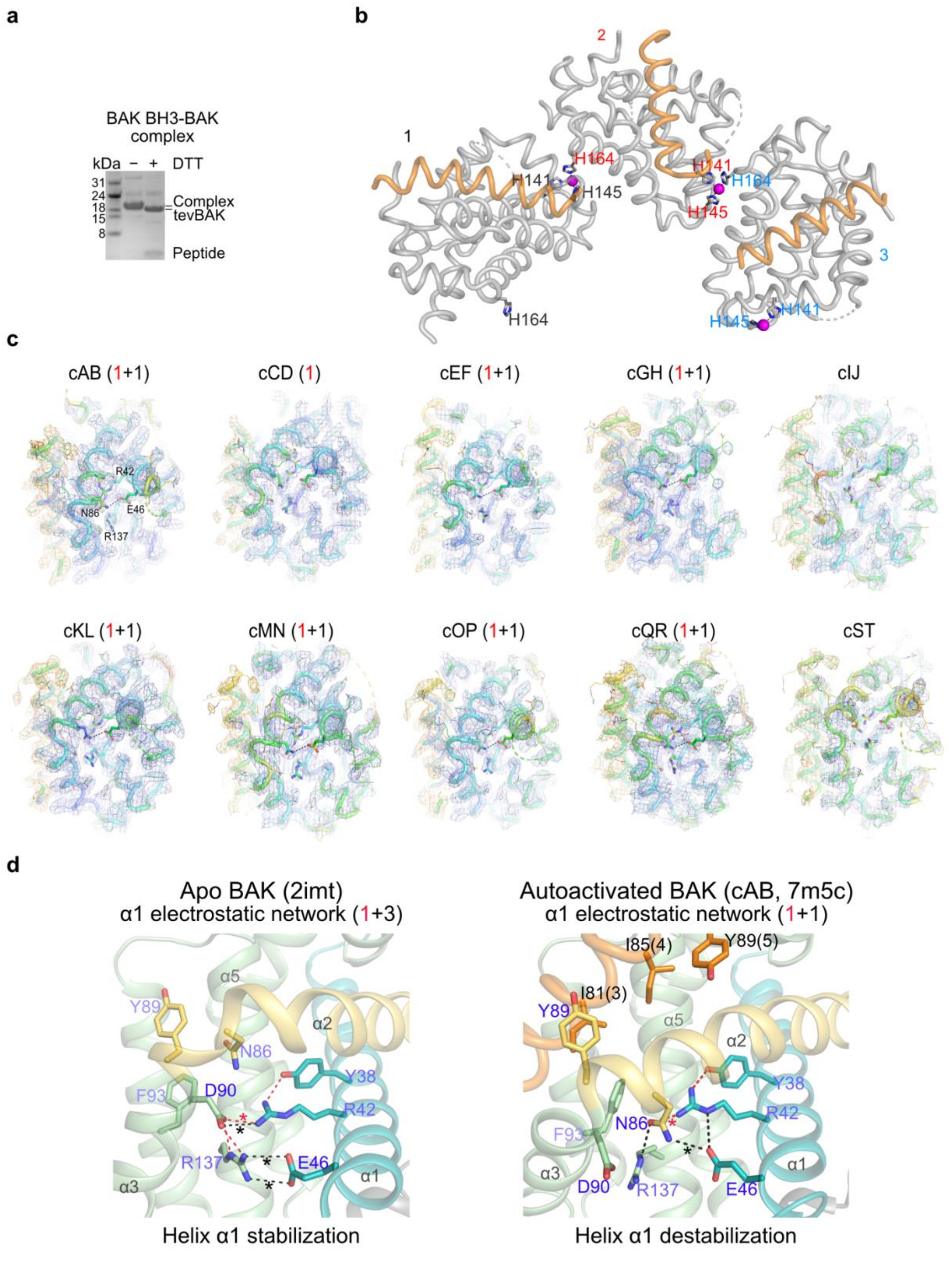

Supplementary Figure 2. Structural details of BAK autoactivation. Related to Figure 1. a, SDS-PAGE of covalent WT BAKGGC-G184C tev BAK complex for crystallography. **b,** Cu^{2+}-mediated crystal packing of three unique BAK BH3-BAK

complexes within the asymmetric unit showing coordination of Cu^{2+} by three His residues. Two of the His are from one complex, and one His is from adjacent complex. **c,** 2Fo-Fc electron density maps contoured at 1σ level over B-factor-colored (blue-to-yellow low-to-high) cartoon representation for 10 unique complexes in the asymmetric unit, all of which exhibit well-defined features of the BAK BH3 peptide and the collapse of the buried electrostatic network involving helix α1. The side chain of Y89 at the C-terminus of BAK BH3 ligand is not observed in the electron density of complexes cCD and cIJ and exhibits poor electron density in complexes cOP and cQR. Hydrogen bonds ≤3.2 Å and ≤3.6 Å are colored red and black, respectively, and are summarized above each complex and in Table S2. Complexes IJ and ST lack well-defined electron density for this electrostatic network. The other complexes exhibit density for the side chain of α2 N86, which hydrogen bonds to α1 R42 and E46, except complex CD in which N86 hydrogen bonds only to R42. α5 R137 no longer stabilizes helix α1 as seen in apo BAK (Figure 1f). **d,** Side-by-side view of the electrostatic network within the protein core at the interface of helices α1, α2, α3, and α5 in apo and autoactivated BAK. Hydrogen bonds ≤3.2 Å (red) and ≤3.6 Å (black) between helix α1 and the rest of the domain identified with * are summarized in brackets and Supplementary Table 3. Corresponding overlaid view is shown in Figure 1f.

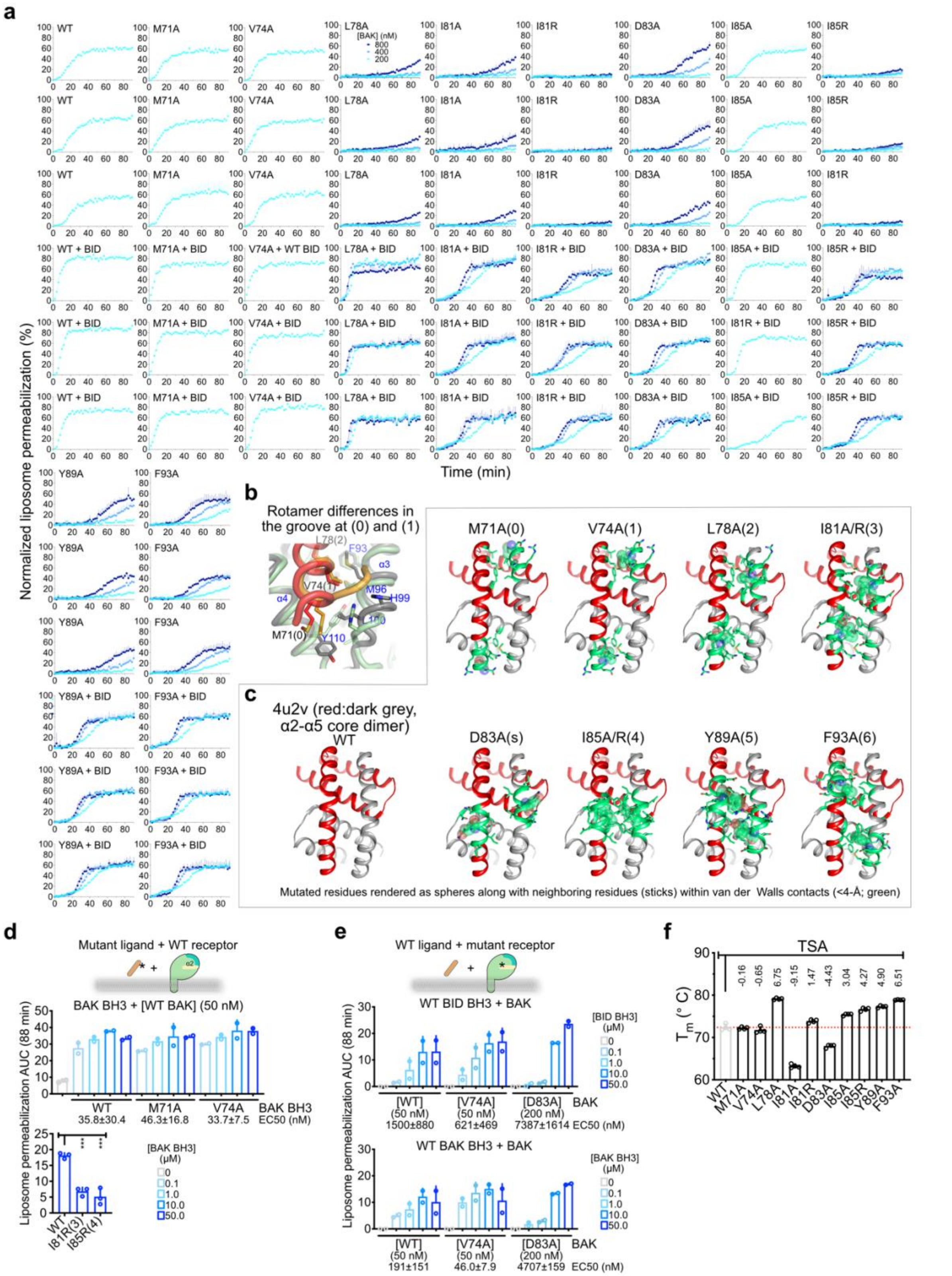

Supplementary Figure 3. Autoactivation and direct activation of BAK use the BH3 and groove. Related to Figure 2, Supplementary Figure 4. a, Liposome permeabilization assays measuring autoactivation (-BID BH3) and direct activation

[+BID BH3 (10 µM)]. Liposome permeabilization is quantified as the percentage of fluorescent dye release normalized relative to permeabilization by 3% CHAPS detergent. Data are presented as mean + SD for n=3 experiments each of n=3 technical replicates. BAK doses are color coded as in panel “L78A”. AUC quantification is shown in Figure 2b. **b,** Cartoon representation of BAK BH3-BAK structure overlaid onto that of α2-α5 core BAK dimer. These structures exhibit heterogeneity of side chain rotamers for Y110, L100, H99, and M96 of the hydrophobic groove in the vicinity of BAK BH3 M71(0) and V74(1). These changes likely do not have major functional relevance given that M71A and V74A mutants behave similar to WT BAK BH3 in BAK activation assays (see Figures 2b, Supplementary Figures 3d, 3e). **c,** Cartoon representation of the α2-α5 core dimer showing the location of tested mutations. Figure 2d summarizes observed and predicted effect of mutations on direct activation, asymmetric BH3-in-groove autoactivation, and symmetric BH3-in-groove α2-α5 core dimerization. **d and e,** Liposome permeabilization assays quantified as AUC of kinetic traces from Supplementary Figure 4 for combination of mutant ligands and WT receptors (e) and WT ligands and mutant receptors (f). Data are presented as mean + SEM of n=3 experiments each of n=3 technical replicates. EC50 values represent mean ± SD from plots of AUC against peptide concentration in Supplementary Figures 4b, 4e. Data for WT BAK activation by WT BID and BAK BH3 are the same in Figure 1D. **f,** Dye-based thermal shift assay (TSA) testing BAK stability at 0.25 mg/mL protein. Melting temperature (T_m) data are represented as mean + SEM for n=4 experiments each of n=4 technical replicates. T_m differences (ºC) relative to BAK are indicated above each bar.

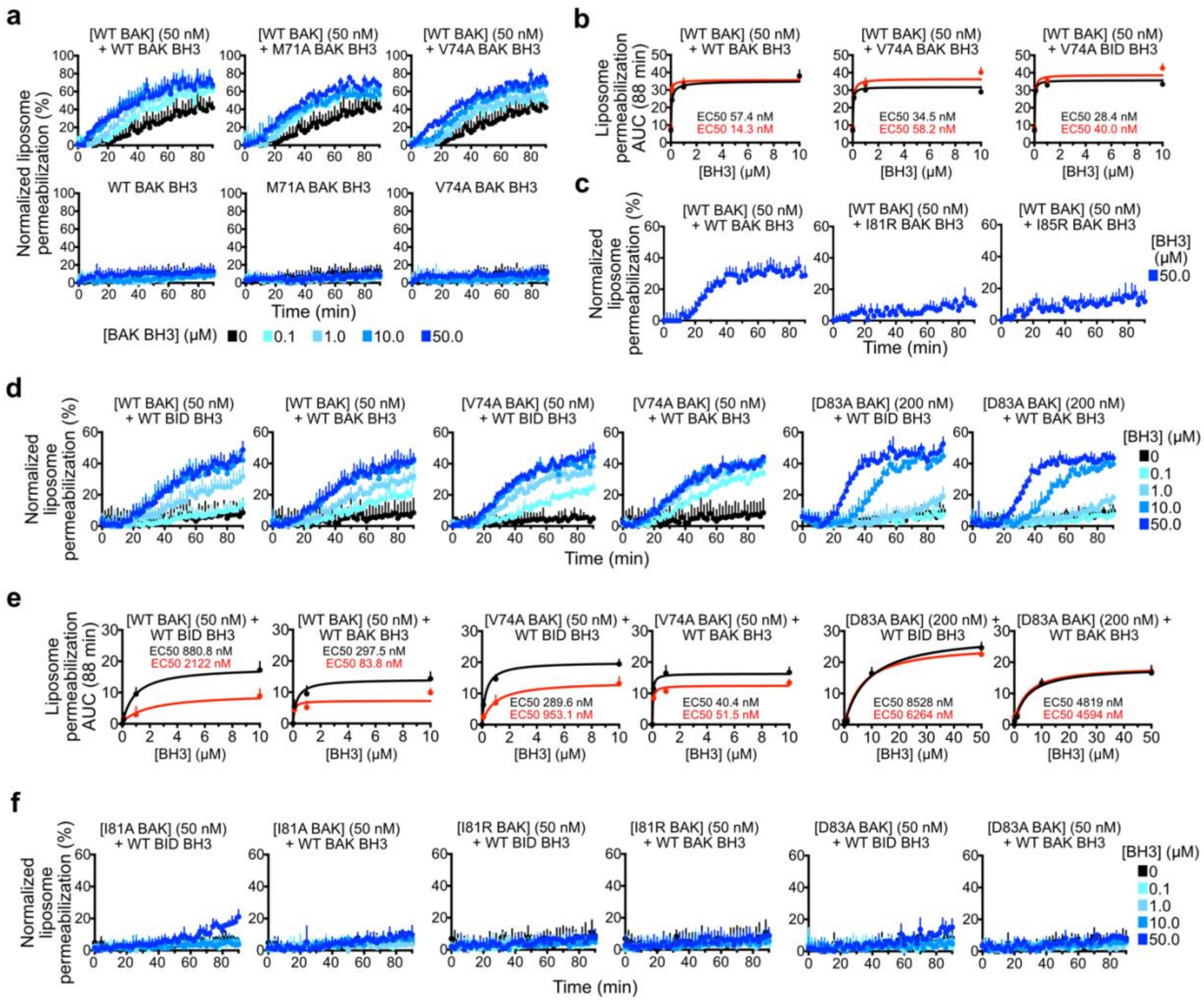

Supplementary Figure 4. Direct activation and autoactivation of BAK with BH3 peptides. Related to Supplementary Figure 3. a and c, Liposome permeabilization assays testing mutant BAK BH3 ligand activation of WT BAK receptor. Liposome permeabilization is quantified as the percentage of fluorescent dye release normalized relative to permeabilization by 3% CHAPS detergent. Data are presented as mean + SD from n=2 experiments each of n=3 technical replicates. AUC quantification is shown in Supplementary Figure 3d and panel (b). **b and e,** Plots of AUC values against peptide dose for liposome permeabilization traces used to calculate EC50 values from panel (a) and (d), respectively. Data in red are for the duplicate experiments.

d and f, Liposome permeabilization assays testing WT BID BH3 and WT BAK BH3 ligand activation of mutant BAK receptor. At 50 nM mutants impaired in autoactivation are resistant to activation (f), but they can be activated at 200 nM. Liposome permeabilization is quantified as the percentage of fluorescent dye release normalized relative to permeabilization by 3% CHAPS detergent. Data are presented as mean + SD from n=2 experiments each of n=3 technical replicates. AUC quantification is shown in Supplementary Figure 3e and panel (e).

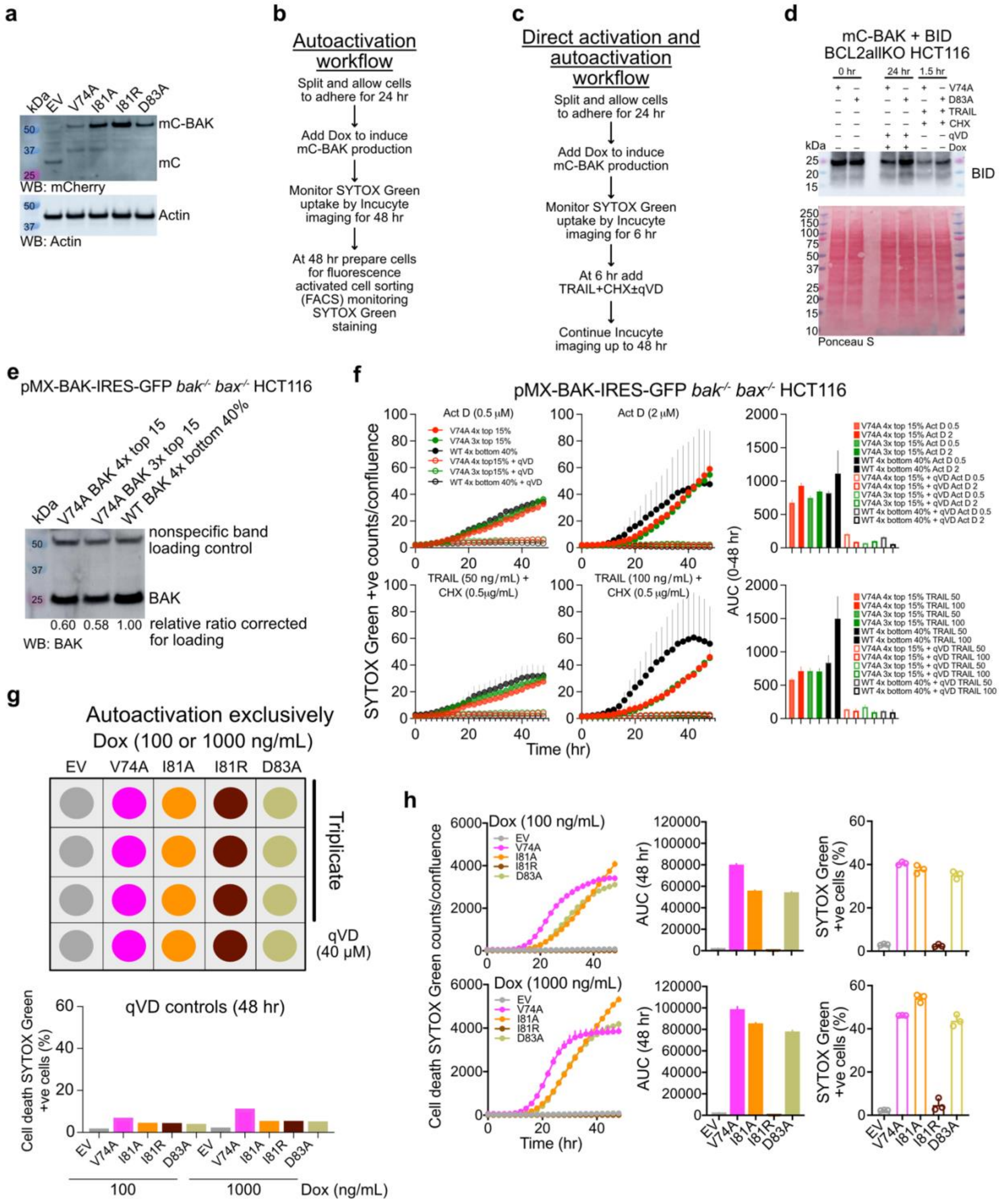

Supplementary Figure 5. Investigation of autoactivation and direct activation of BAK in cells. Related to Figure 3. a, Representative immunoblot of BCL2allKO HCT116 cells expressing mCherry (mC) empty vector (EV) or mutant mC-BAK after treatment with Dox (1000 ng/mL) and qVD (40 µM) for 36 hr from n=2 independent experiments. **b,** Workflow to engage autoactivation of BAK in BCL2allKO HCT116 cells reconstituted with doxycycline (Dox)-inducible mC-BAK. **c,** Workflow to engage direct

activation and autoactivation of BAK in BCL2allKO HCT116 cells reconstituted with BID and doxycycline (Dox)-inducible mC-BAK. **d,** Representative immunoblot for BID and sypro red staining of BCL2allKO HCT116 cells expressing V74A and D83A mC-BAK and BID from n=2 independent experiments. **e,** Representative immunoblot of *bak*$^{-/-}$ *bax*$^{-/-}$ HCT116 cells constitutively expressing WT and V74A BAK using pMX-BAK-IRES-GFP from n=2 independent experiments. GFP positive cells were sorted three and four times to achieve similar expression levels. WT BAK expresses robustly in these cells but V74A BAK levels do not achieve higher levels upon additional sorting. **f,** Apoptosis of BAK-expressing cells in (d) initiated by actinomycin D (Act D) and combination of human TRAIL and cycloheximide (CHX). Higher BAK levels may explain the faster kinetics by WT BAK at 200 ng/mL TRAIL. Data are presented as mean + SD for one representative of n=2 experiments each of n=3 technical replicates. **g**, Experimental setup and inhibition of cell death by caspase inhibitor qVD related to data in (h). **h,** Autoactivation-dependent cell death of BCL2allKO HCT116 reconstituted with mCherry-BAK (mC-BAK) monitored for 48 hr by IncuCyte imaging uptake of cell-impermeable dye SYTOX Green. AUC for kinetic traces is included. At 48 hr, we quantified percentage of SYTOX Green +ve cells (%) by fluorescence activated cell sorting (FACS). Data are presented as mean + SD from one of n=3 experiments each of n=3 technical replicates.

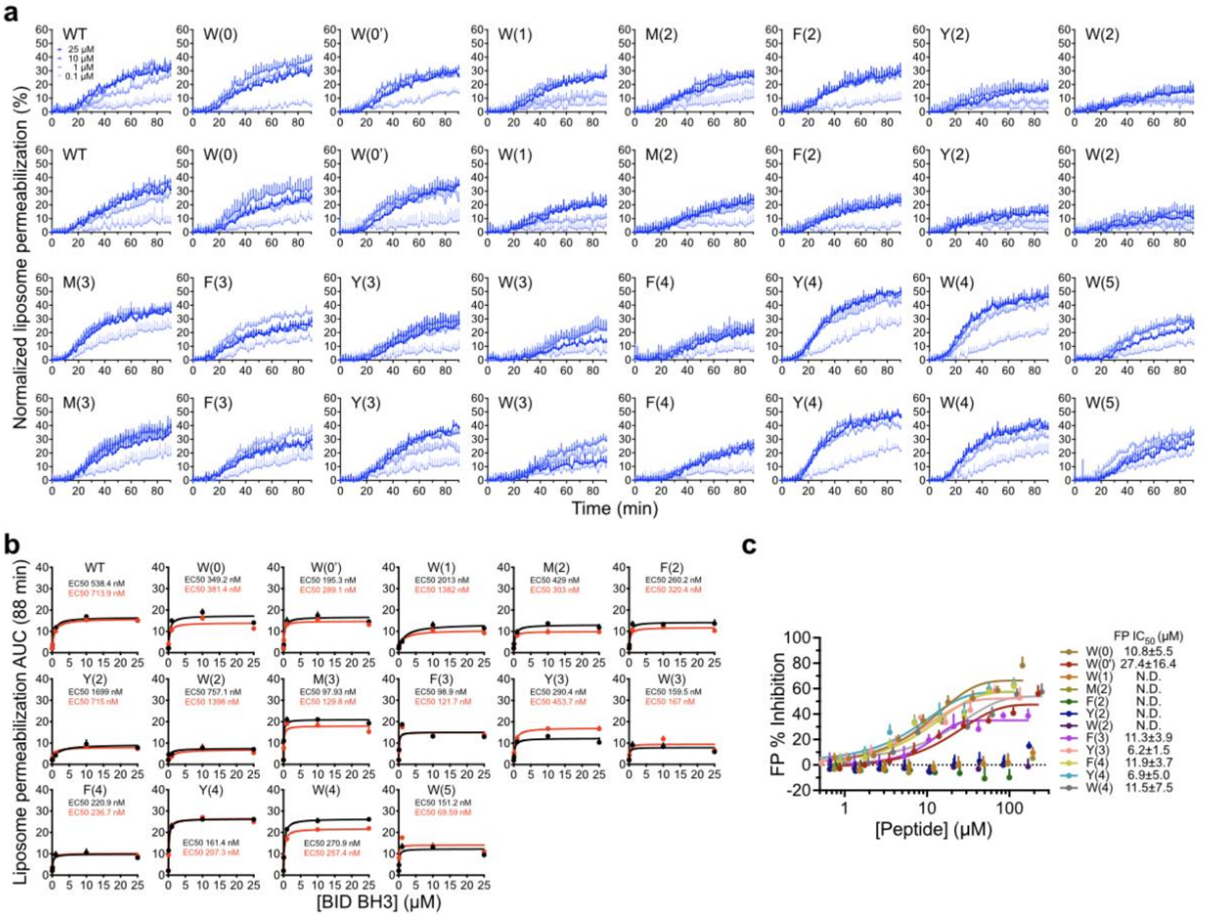

Supplementary Figure 6. Direct BAK activation by monosubstituted BID-like BH3 peptides. Related to Figure 4. a, Kinetic traces of liposome permeabilization by BAK (50 nM) and BH3 peptides in Figure 4b. Peptide doses are color coded as in panel "WT". Liposome permeabilization is quantified as the percentage of fluorescent dye release normalized relative to permeabilization by 3% CHAPS detergent. Data are presented as mean + SD of n=2 experiments each of n=3 technical replicates. **b,** Plots of AUC values against peptide dose for liposome permeabilization traces in panel (a) used to calculate EC50 values. **c,** Competitive fluorescence polarization assay measuring displacement of BID SAHB-fluorescein peptide from GST-BAK by BH3 peptides from Figure 4b. Data is presented as mean ± SD from n=9 experiments, except n=7 for W(1) peptide.

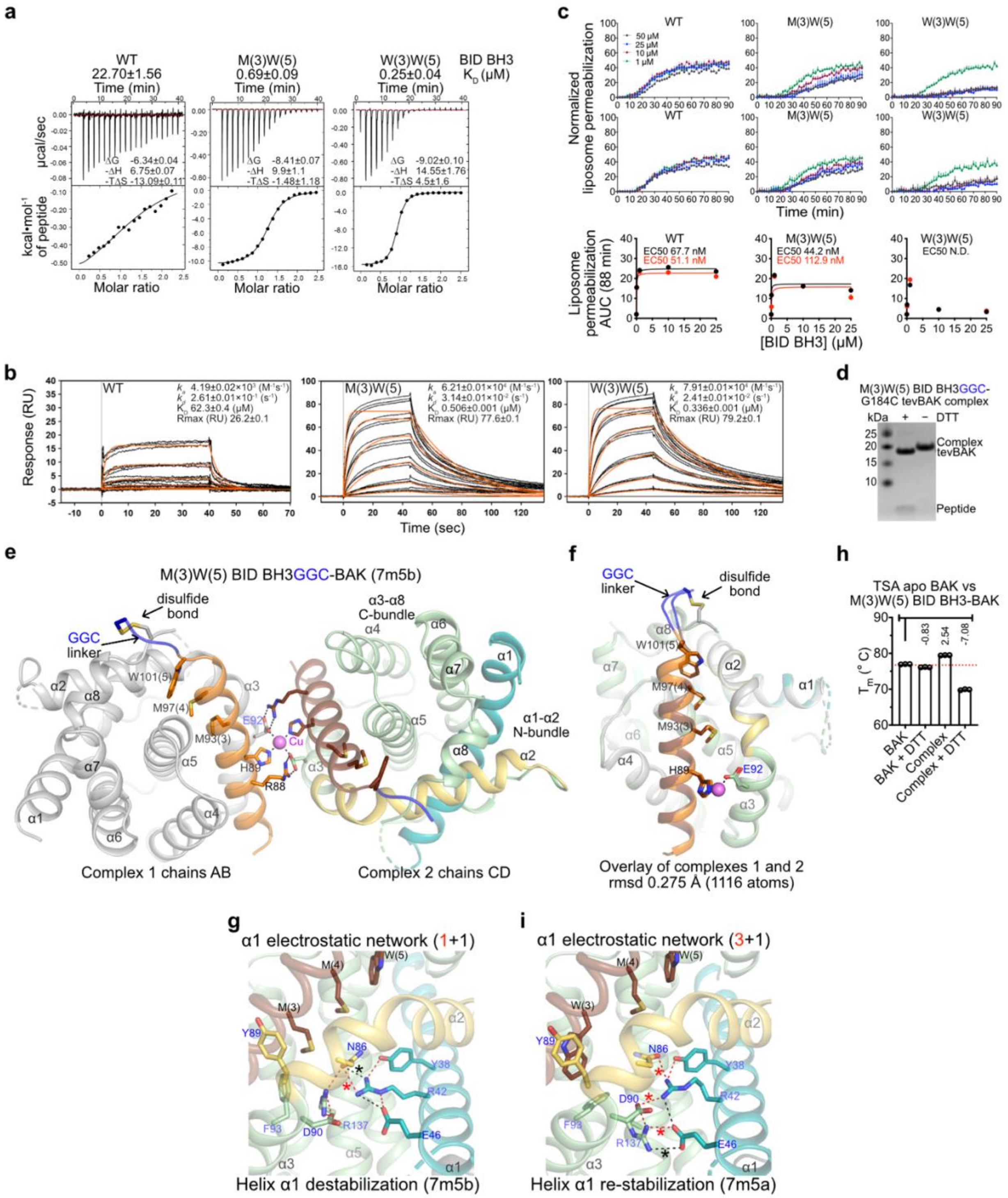

Supplementary Figure 7. BAK modulation by high-affinity BID-like BH3 ligands. Related to Figure 5. a, ITC traces (top) and binding isotherms (bottom) for 50 µM BAK titrated with BH3 peptides in Figure 5a for one representative of n=2 experiments. **b,** Surface plasmon resonance (SPR) sensorgrams for BAK titrated with the BH3 peptides in Figure 5a. MEAS-BAK-H_6 was immobilized to nickel-nitrilotriacetic acid (Ni-NTA) sensor chip. Theoretical profile (orange) are plotted over raw data (black). **c,** Kinetic traces of liposome permeabilization by 50 nM BAK + BH3 peptides in Figure 5a.

Liposome permeabilization is quantified as the percentage of fluorescent dye release normalized relative to permeabilization by 3% CHAPS detergent. Data are presented as mean + SD of n=3 experiments each of n=3 technical replicates. AUC quantification is presented below. **d,** SDS-PAGE of covalent M(3)W(5) BID BH3GGC-G184C tev BAK complex for crystallography. **e,** Cartoon representation of Cu^{2+}-mediated dimer structure in the asymmetric unit of the M(3)W(5) BID BH3GGC-G184C tevBAK crystal. Only one of the complexes exhibits continuous density for the GGC linker and disulfide bond to G184C of tevBAK. **f,** Overlay of the asymmetric unit complexes in (e) indicates very similar structures, suggesting that the GGC linker does not induce gross structural changes.

g and i, Electrostatic network of helices α1, α2, α3, and α5 (bottom) for directly activated complex (g) and inactivated complex (i) of BAK bound to BID-like BH3 peptides. The electrostatic network is destabilized and re-stabilized in these complexes, respectively, compared to that observed in apo BAK (Figures 5e, 5g, S2d). Hydrogen bonds ≤3.2 Å (red) and ≤3.6 Å (black) between helix α1 and the rest of the domain marked by asterisks are summarized in brackets and Table S2. **h,** Dye-free nano Differential Scanning Fluorimetry TSA of apo BAK and the activating M(3)W(5) BID BH3GGC-BAK complex at 15 µM. T_m data are presented as mean + SD from n=3 experiments each of n=1 technical replicates. T_m differences (ºC) relative to BAK are indicated above each bar.

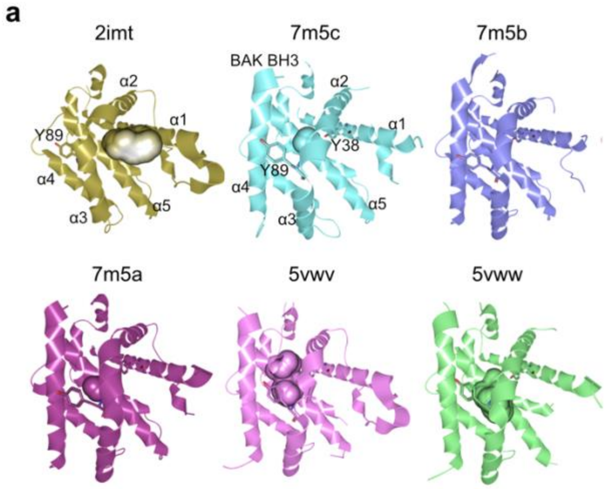

Structure ID	Pocket Volume[1] (Å^3)
2imt (Apo)	
7m5c (BAK BH3)	69.8
7m5b (M3W5 BID BH3)	None identified in the vicinity of Y89
7m5a (W3W5 BID BH3)	64.5
5vwv (BIMRT BH3)	313.9
5vwv (BIMRT BH3)	275.3

Apo
Active
Inactive

[1] BAK structures were analyzed for pockets/cavities with CAVER2.0 software using a 2-Å probe size. Volumes were estimated using ≥5,000 samples to improve precision.

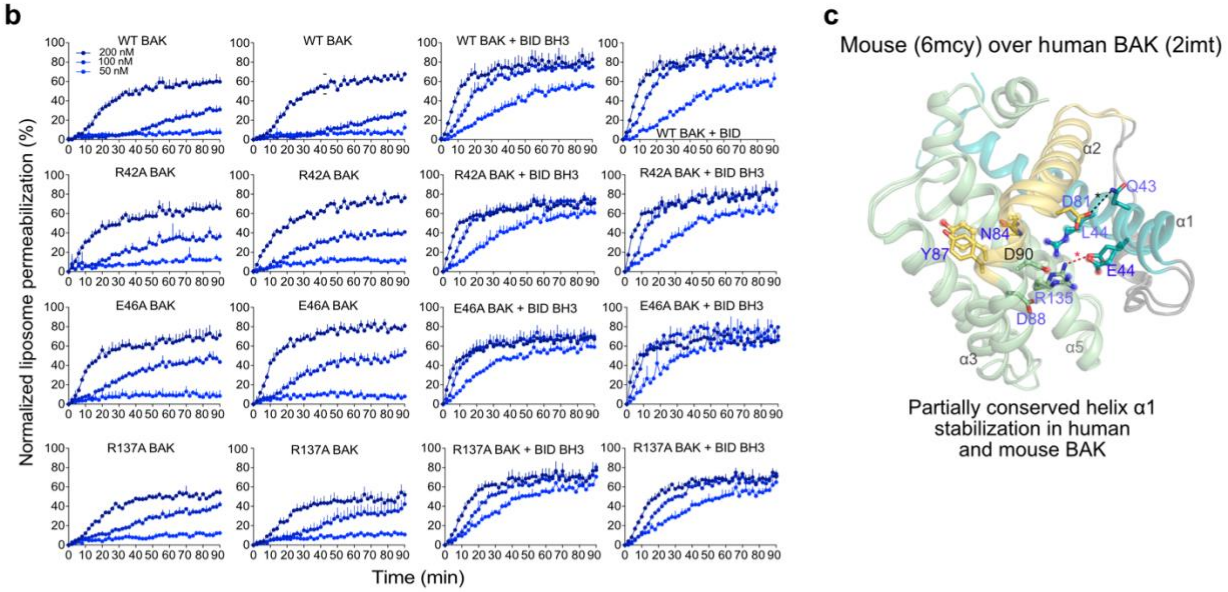

Supplementary Figure 8. BH3-induced cavities in BAK do not correlate with its activation. Related to Figure 7. a, Cavities at the activation groove of BAK were calculated for apo BAK, and activated and inactivated BH3:BAK complexes with CAVER Analyst 2.0[44]. Solvent molecules were removed for this analysis. **b,** Kinetic traces of liposome permeabilization by WT and mutant BAK ± WT BID BH3 peptide (10 µM). Liposome permeabilization is quantified as the percentage of fluorescent dye release normalized relative to permeabilization by 3% CHAPS detergent. Data are presented as mean + SD for n=3 experiments each of n=3 technical replicates. BAK doses are color coded in panel WT BAK. **c**, Overlay of mouse and human apo BAK structures reveals an alternative electrostatic network stabilizing helix 1 comprised of conserved salt bridge E44–R135, buried in a similar location as in human BAK (E46–R137); and solvent-exposed salt bridge Q43–D81, replacing the buried one (R42–D90) in human BAK. A different arrangement of helix 3 places mouse D88 away from its position in human BAK (D90).

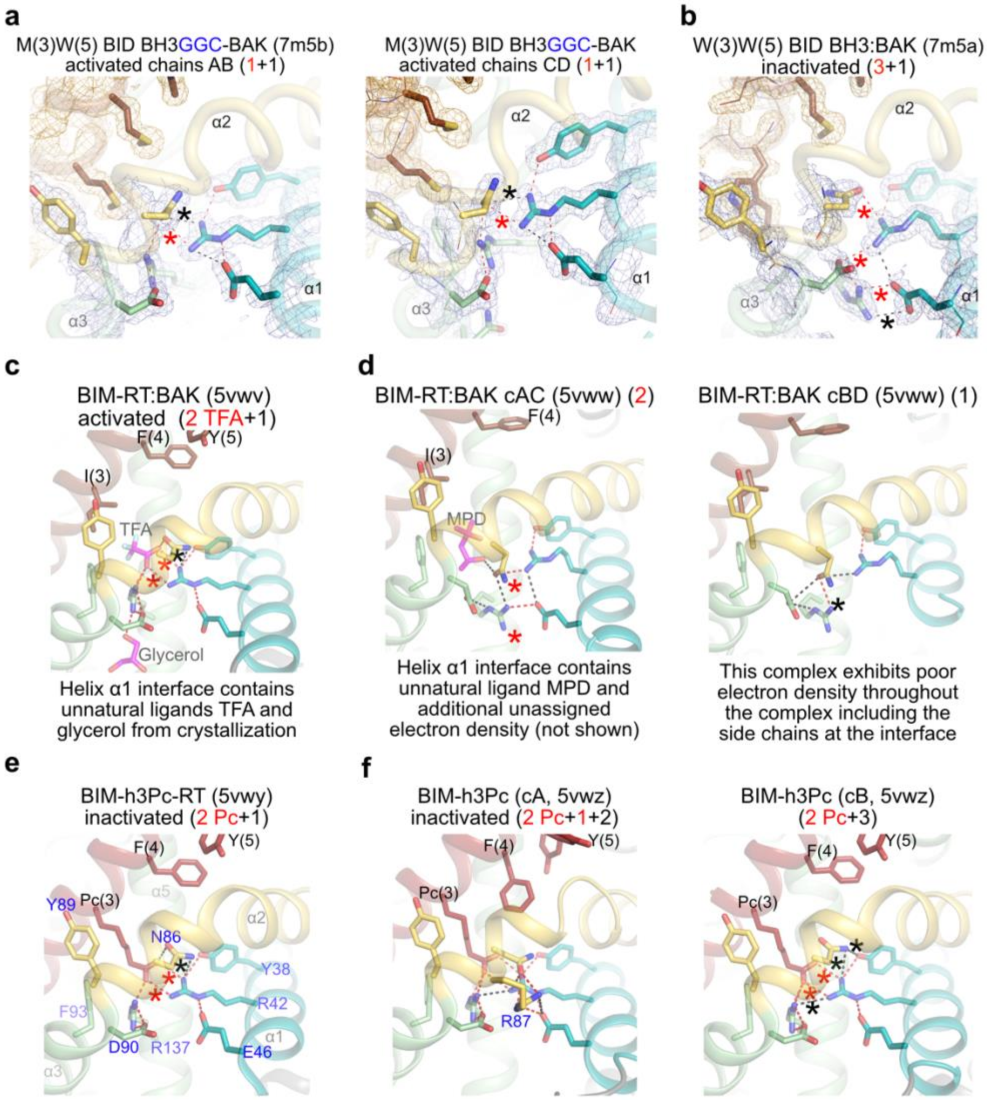

Supplementary Figure 9. Buried electrostatic network regulates BAK activation and inactivation in BH3 ligand-dependent manner. Related to Figure 6. a and b, 2Fo-Fc electron density maps contoured at 1σ level over cartoon representation for BH3 peptides and buried helix α1 electrostatic networks of two complexes found in the asymmetric unit of M(3)W(5) BID BH3GGC-G184C tevBAK crystal (a) and W(3)W(5) BID BH3:tevBAK complex (b). **c and d,** Cartoon representation of buried electrostatic network of activated BAK complexes with BIM-RT BH3 (c and d). **e and f,** Cartoon representation of buried electrostatic networks of inactivated “molecular glue” complexes of BAK with BIM-like BH3 peptide. Hydrogen bonds ≤3.2 Å (red) and ≤3.6 Å (black) connecting helix α1 to the rest of the domain are marked with * and summarized in brackets and Supplementary Table 3.

Supplementary Figure 2a

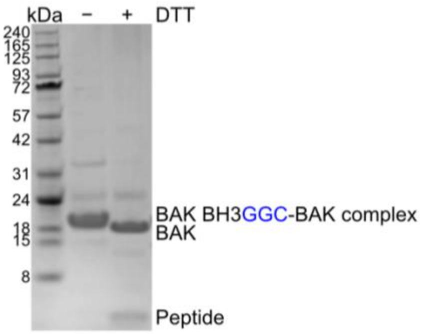

Supplementary Figure 7d

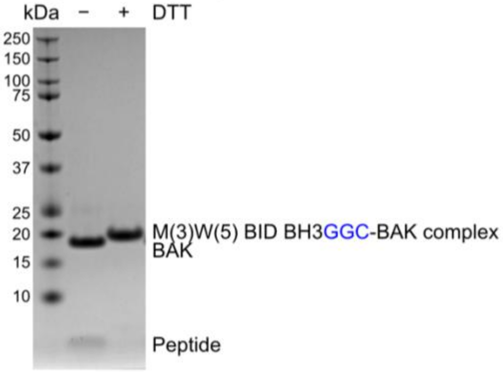

Supplementary Figure 5a

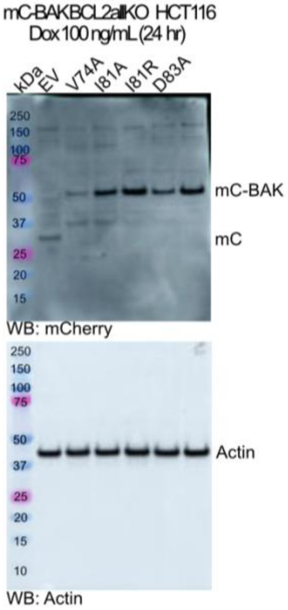

Supplementary Figure 5d

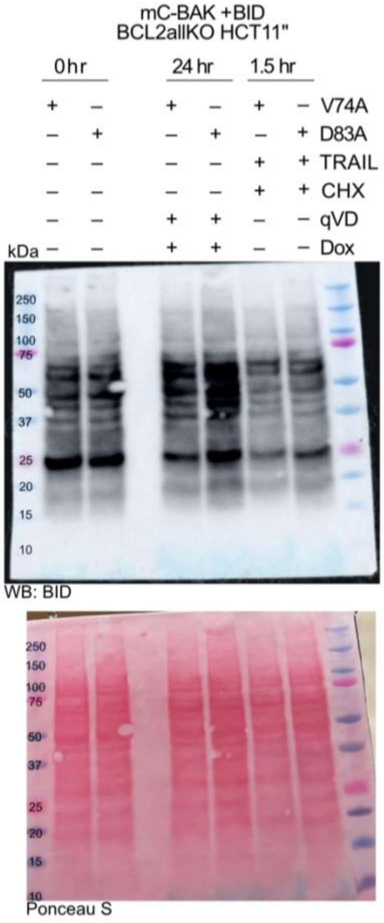

Supplementary Figure 5e

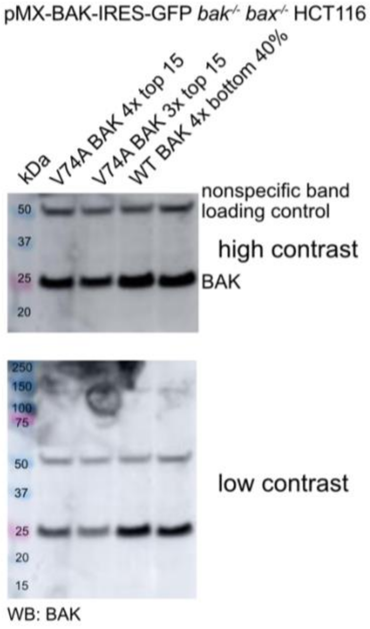

Uncropped gels and blots from Supplementary Figures also found in the Source Data File.
