# Supplementary material for: Structural basis of BAK activation in mitochondrial apoptosis initiation
Source: Nat Commun. 2022 Jan 11;13:250. doi: 10.1038/s41467-021-27851-y (PMC8752837; doi:10.1038/s41467-021-27851-y)
Supplement: Supplementary file 3 — Description of Additional Supplementary Files [file 41467_2021_27851_MOESM3_ESM.pdf]

Description of Additional Supplementary Files

File Name: Supplementary Movie 1
Description: **BH3 ligand induced conformational changes in BAK.** Morphing of the helix α1-stabilizing electrostatic network between apo and BH3 ligand bound conformations were generated in PyMOL.

File Name: Supplementary Movie 2
Description: **Direct activation and autoactivation cooperate to amplify BAK activation signaling.**
