# Supplementary material for: Structural basis of BAK activation in mitochondrial apoptosis initiation
Source: Nat Commun. 2022 Jan 11;13:250. doi: 10.1038/s41467-021-27851-y (PMC8752837; doi:10.1038/s41467-021-27851-y)
Supplement: Supplementary file 6 — Reporting Summary [file 41467_2021_27851_MOESM6_ESM.pdf]

nature research

Corresponding author(s): Dr. Tudor Moldoveanu

Last updated by author(s): Dec 9, 2021

Reporting Summary

Nature Research wishes to improve the reproducibility of the work that we publish. This form provides structure for consistency and transparency in reporting. For further information on Nature Research policies, seeAuthors & Referees and theEditorial Policy Checklist .

Statistics

For all statistical analyses, confirm that the following items are present in the figure legend, table legend, main text, or Methods section.

n/a	Confirmed	
☐	☒	The exact sample size (*n*) for each experimental group/condition, given as a discrete number and unit of measurement
☐	☒	A statement on whether measurements were taken from distinct samples or whether the same sample was measured repeatedly
☐	☒	The statistical test(s) used AND whether they are one- or two-sided<br>*Only common tests should be described solely by name; describe more complex techniques in the Methods section.*
☐	☒	A description of all covariates tested
☐	☒	A description of any assumptions or corrections, such as tests of normality and adjustment for multiple comparisons
☐	☒	A full description of the statistical parameters including central tendency (e.g. means) or other basic estimates (e.g. regression coefficient) AND variation (e.g. standard deviation) or associated estimates of uncertainty (e.g. confidence intervals)
☐	☒	For null hypothesis testing, the test statistic (e.g. *F*, *t*, *r*) with confidence intervals, effect sizes, degrees of freedom and *P* value noted<br>*Give P values as exact values whenever suitable.*
☒	☐	For Bayesian analysis, information on the choice of priors and Markov chain Monte Carlo settings
☒	☐	For hierarchical and complex designs, identification of the appropriate level for tests and full reporting of outcomes
☒	☐	Estimates of effect sizes (e.g. Cohen's *d*, Pearson's *r*), indicating how they were calculated

Our web collection on statistics for biologists contains articles on many of the points above.

Software and code

Policy information about availability of computer code

Data collection	*Provide a description of all commercial, open source and custom code used to collect the data in this study, specifying the version used OR state that no software was used.*
Data analysis	X-ray data collection was performed using the beamline control program (e.g. SER-CAT control program)<br>X-ray data analyzed using HKL2000 v719.2, REFMAC v5.8.0238, XDS vMar 15, 2019, Phenix 1.17.1, Coot v0.8.9.2 EL, MacPyMOL v1.7.6.3<br>Cell Death data analysed using IncuCyte Zoom v2016A, IncuCyte ZOOM 2016A Rev2 v20161.1.5932.22771, FlowJo X v10.0.7r2<br>Competitive fluorescence polarization assay data analyzed using Robust Investigation of Screening Experiments (RISE) developed at St. Jude Children's Hospital on the Pipeline Pilot platform (Accelrys, v.8.5.0)<br>Liposome permeabilization analyzed using Microsoft Word Excel 2013, GraphPad Prism v8.0a<br>Structure cavity analysis CAVER Analyst 2.0

For manuscripts utilizing custom algorithms or software that are central to the research but not yet described in published literature, software must be made available to editors/reviewers. We strongly encourage code deposition in a community repository (e.g. GitHub). See the Nature Research guidelines for submitting code & software for further information.

Data

Policy information about availability of data

All manuscripts must include a data availability statement. This statement should provide the following information, where applicable:

- Accession codes, unique identifiers, or web links for publicly available datasets
- A list of figures that have associated raw data
- A description of any restrictions on data availability

The accession numbers for the atomic coordinates and structure factors deposited are PDB: 7m5a, 7m5b, 7m5c

Field-specific reporting

Please select the one below that is the best fit for your research. If you are not sure, read the appropriate sections before making your selection.

- [x] Life sciences
- [ ] Behavioural & social sciences
- [ ] Ecological, evolutionary & environmental sciences

For a reference copy of the document with all sections, see nature.com/documents/nr-reporting-summary-flat.pdf

Life sciences study design

All studies must disclose on these points even when the disclosure is negative.

Sample size	We have previously established that data presented in our manuscript exhibit statistically significant differences when n>=2 experiments are performed in triplicate. The liposome permeabilization data are very reproducible especially when performed using the same liposome preparation. Therefore we typically performed all of our experiments with n=2 or n=3 in triplicate or quadruplicate.
Data exclusions	For the FP experiments we have excluded the data obtained at high peptide concentration as we noticed severe peptide precipitation under those conditions; Occasionally one of n=3 experiments exhibits a much greater difference in trends compared to the other two in which case we have excluded that experiment. Sometimes we exclude a repeat within a triplicate for one of the representative experiments as an outlier.
Replication	For individual experiments data is plotted as mean of triplicate or quadruplicate measurements with error bars of standard deviation (SD). Bar graph data is combined from two to three independent experiments as average and standard error of the mean (SEM). Statistical analyses summarized in each figure analyzed by one-way ANOVA using the Tukey-Kramer method for multiple comparisons with two-tailed or multiplicity adjusted.
Randomization	For liposome permeabilization assays, which cannot be done at the same time (i.e. multiple plates are usually analyzed over the course of 1-2 days), we compare every condition tested against the WT BAK control and 3% CHAPS release using the same liposome preparation. For cell death analysis we have compared all the conditions side-by-side with V74A BAK, which acted as the surrogate WT BAK. For FP and TSA analyses we performed all the experiments side-by-side in the same plate. Therefore, we do not believe non-random patterns have affected our analysis
Blinding	All of our assays are repeated multiple times being extensively tested, well-behaved and reproducible and have not required blinding based on vast amount of literature in field.

Reporting for specific materials, systems and methods

We require information from authors about some types of materials, experimental systems and methods used in many studies. Here, indicate whether each material, system or method listed is relevant to your study. If you are not sure if a list item applies to your research, read the appropriate section before selecting a response.

Materials & experimental systems

n/a	Involved in the study	
[ ]	[x]	Antibodies
[ ]	[x]	Eukaryotic cell lines
[x]	[ ]	Palaeontology
[x]	[ ]	Animals and other organisms
[x]	[ ]	Human research participants
[x]	[ ]	Clinical data

Methods

n/a	Involved in the study	
[x]	[ ]	ChIP-seq
[ ]	[x]	Flow cytometry
[x]	[ ]	MRI-based neuroimaging

Antibodies

Antibodies used	Anti-actin monoclonal antibody from Millipore Sigma, Cat# MAB1501;<br>Anti-BAK (Ab-1) mouse monoclonal antibody from Calbiochem, Cat# AM03<br>Anti-BAK (3814s) rabbit polyclonal antibody from Cell Signaling, Cat# 3814s<br>Anti-human BID monoclonal antibody from Santa Cruz, Cat# SC-56025<br>Anti-mCherry monoclonal Antibody (16D7) from Thermofisher, Cat# M11217<br>Anti-cytochrome c (7H8) mouse monoclonal antibody from Santa Cruz, Cat# sc-13560<br>Anti-rat IgG, Horseradish Peroxidase whole antibody (goat) from GE Lifescience, Cat# NA935<br>Anti-rabbit ECL antibody from GE Lifescience, Cat# NA934<br>Anti-mouse IgG ECL secondary antibody (sheep) from GE Lifescience, Cat# NA931
Validation	All antibodies are validated by the vendor and by many researchers that have published studies with these.<br>Anti-actin monoclonal antibody from Millipore Sigma, Cat# MAB1501; https://www.emdmillipore.com/US/en/product/Anti-Actin-Antibody-clone-C4,MM_NF-MAB1501; It has a rating MQ100 by the vendor; This is one of the best antibodies for purchase

we used in our lab routinely as loading control.
Anti-BAK (Ab-1) mouse monoclonal antibody from Calbiochem, Cat# AM03; https://www.emdmillipore.com/US/en/product/Anti-Bak-Ab-1-Mouse-mAb-TC-100,EMD_BIO-AM03; used in Chittenden, T., et al. 1995. Nature 374, 733; Farrow, S.N., et al. 1995. Nature 374, 731; Kiefer, M.C., et al. 1995. Nature 736; Reed, J.C. 1994. J. Cell Biol. 124, 1; Korsmeyer, S.J., et al. 1993. N. Semin. Cancer Biol. 4, 327. We use this Ab routinely in the lab.
Anti-BAK (3814s) rabbit polyclonal antibody from Cell Signaling, Cat# 3814s; https://www.cellsignal.com/products/primary-antibodies/bak-antibody/3814; used in Gross, A. et al. (1999) Genes Dev. 13, 1899-1911; Wei, M.C. et al. (2001) Science 292, 727-730; Zong, W.X. et al. (2001) Genes Dev. 15, 1481-1486; Degenhardt, K. et al. (2002) J. Biol. Chem. 277, 14127-14134; Wei, M.C. et al. (2000) Genes Dev. 14, 2060-2071.
Anti-human BID monoclonal antibody from Santa Cruz, Cat# SC-56025; https://www.scbt.com/p/bid-antibody-5c9; 34 citations have been linked to the manufacturer's website.
Anti-mCherry monoclonal Antibody (16D7) from Thermofisher, Cat# M11217; https://www.thermofisher.com/antibody/product/M11217.html?ef_id=CjwKCAiA78aNBhAlEiwA7B76p_TdhtM4xtz8IELtyjO5ZgCmz3PmH2-q5mOkVW07jRBCZWBigvLIUBoCNrcQAvD_BwE:G:s&s_kwcid=AL!3652!3!459737518508!!!g!!&cid=bid_pca_aup_r01_co_cp1359_pjt0000_bid00000_0se_gaw_dy_pur_con&gclid=CjwKCAiA78aNBhAlEiwA7B76p_TdhtM4xtz8IELtyjO5ZgCmz3PmH2-q5mOkVW07jRBCZWBigvLIUBoCNrcQAvD_BwE; 81 references are found on manufacturer's website
Anti-cytochrome c (7H8) mouse monoclonal antibody from Santa Cruz, Cat# sc-13560; this antibody was used during the revisions but related data have not been included in the final manuscript.
Anti-rat IgG, Horseradish Peroxidase whole antibody (goat) from GE Lifescience, Cat# NA935; this secondary antibody has been validated extensively in our lab
Anti-rabbit ECL antibody from GE Lifescience, Cat# NA934; this secondary antibody has been validated extensively in our lab
Anti-mouse IgG ECL secondary antibody (sheep) from GE Lifescience, Cat# NA931; this secondary antibody has been validated extensively in our lab

Eukaryotic cell lines

Policy information about cell lines

Cell line source(s)

allBCL-2KO HCT116 colorectal carcinoma cell line genetically modified to lack the BCL-2 family repertoire (17 genes)
bak-/- bax-/- HCT116 cells; Neither cell line is commercially available

Authentication

Authentication for allBCL-2 HCT116 cell lines was done by the Xu Luo lab from University of Nebraska, who created them (O'Neill KL et al. 2016 Genes Dev 30:973-988.
Authentication of bak-/- bax-/- HCT116 cells was done by immunobloting. Cells were from Wang and Youle Oncogene 2012 31:3177-3189.

Mycoplasma contamination

We quarantined allBCL-2KO HCT116 and treated them with mycoplasma reagents to ensure they are mycoplasma free by PCR.

Commonly misidentified lines
(See ICLAC register)

No commonly misidentified lines were used in this study.

Flow Cytometry

Plots

Confirm that:

- [x] The axis labels state the marker and fluorochrome used (e.g. CD4-FITC).
- [x] The axis scales are clearly visible. Include numbers along axes only for bottom left plot of group (a 'group' is an analysis of identical markers).
- [ ] All plots are contour plots with outliers or pseudocolor plots.
- [x] A numerical value for number of cells or percentage (with statistics) is provided.

Methodology

Sample preparation

This standard cell death assay involves trypsinization of adherent cells, wash in PBS, and direct analysis for SYTOX Green positivity

Instrument

FACSCalibur (BD Biosciences)

Software

FlowJo X

Cell population abundance

The intact cell population was identified by FSC and SSC light scattering measurements; Within that population the SYTOX Green population is clearly distinguished representing dying cells. We observed more than 40% SYTOX Green positive cells for the active BAK mutants in cells undergoing apoptosis.

Gating strategy	Gating first on the intact cell population according to FSC vs SSC we perform inspection of a histogram of this population based on FL1 (SYTOX Green) and set the gate to distinguish alive cell and those with compromised plasma membrane integrity (dead cells)

☒ Tick this box to confirm that a figure exemplifying the gating strategy is provided in the Supplementary Information.
